# Foot Pronation Contributes to Altered Lower Extremity Loading After Long Distance Running

**DOI:** 10.3389/fphys.2019.00573

**Published:** 2019-05-22

**Authors:** Qichang Mei, Yaodong Gu, Liangliang Xiang, Julien S. Baker, Justin Fernandez

**Affiliations:** ^1^Faculty of Sports Science, Ningbo University, Ningbo, China; ^2^Research Academy of Grand Health, Ningbo University, Ningbo, China; ^3^Auckland Bioengineering Institute, University of Auckland, Auckland, New Zealand; ^4^Institute for Clinical Exercise and Health Science, University of the West of Scotland, Paisley, United Kingdom; ^5^Department of Engineering Science, University of Auckland, Auckland, New Zealand

**Keywords:** foot posture, pronation, knee, ankle, contact force, OpenSim, statistical parametric mapping

## Abstract

This study presents an investigation of the changes in foot posture, joint kinematics, joint moments and joint contact forces in the lower extremity following a 5 k treadmill run. A relationship between knee and ankle joint loading and foot posture index (FPI) is developed. Twenty recreational male heel-strike runners participated in this study. All participants had a history of running exercise and were free from lower extremity injuries and foot deformities. Foot posture was assessed from a six-item FPI to quantitatively classify high supination to high pronation foot poses. The FPI is scored using a combination of observations and foot palpations. The three-dimensional marker trajectories, ground reaction force and surface electromyography (EMG) were recorded at pre and post-gait sessions conducted over-ground and 5 k running was conducted on a treadmill. Joint kinematics, joint moments and joint contact forces were computed in OpenSim. Simulated EMG activations were compared against experimental EMG to validate the model. A paired sample *t*-test was conducted using a 1D statistical parametric mapping method computed temporally. Hip joint moments and contact forces increased during initial foot contact following 5 k running. Knee abduction moment and superior-inferior knee contact force increased, whereas the knee extension moment decreased. Ankle plantarflexion moment and ankle contact forces increased during stance. FPI was found to be moderately correlated with peak knee and ankle moments. Recreational male runners presented increased static foot pronation after 5 k treadmill running. These findings suggest that following mid distance running foot pronation may be an early indicator of increased lower limb joint loading. Furthermore, the FPI may be used to quantify the changes in knee and ankle joint moments.

## Introduction

Long distance running has increased in popularity (van Gent et al., 2007; Hulme et al., 2017) due to practicality in many environments, low cost, and links to preventing health issues (Mei et al., 2018). Extensive running participation may lead to increased running-related injuries (RRI) reported as 2.5–33.0 injuries per 1000 h of running (Videbæk et al., 2015; Hulme et al., 2017) with up to 79.3% RRI reported at the knee joint (van Gent et al., 2007). The human foot, as the primary interface with our environment, presents morphological and postural changes following prolonged running, which is a key intrinsic factor contributing to RRI (Barnes et al., 2008; Nigg, 2011; Nigg et al., 2015; Mei et al., 2018). A 6-item scale (foot posture index, FPI) was previously developed and validated to define foot postures including high supination, supination, neutral, pronation and high pronation in multiple planes and anatomical segments under static palpation measurements and clinical settings (Redmond et al., 2006). This FPI may play a role as a low-cost assessment of foot postures without requiring a lab or imaging evaluation.

Over 90% of recreational marathon runners adopt a heel-strike style (Larson et al., 2011). This is associated with a drop in foot arch following long distance running (Mei et al., 2018), which is consistent with a recent finding reporting reduced arch ratio and foot pronation (Fukano et al., 2018). A recent study reported that competitive runners exhibited higher local dynamic foot stability quantified by the “Maximal Lyapunov Exponent” compared with recreational runners during an exhaustive 5 k run (Hoenig et al., 2019). A high-intensity treadmill run exhibited symmetry in step length, step frequency, contact time, flight time, maximum force and impulse but asymmetry in impact force (at 5 k), and flight time together with impact force (at 7.5–10 k) (Hanley and Tucker, 2018). Skeletal joint work shifted proximally from the ankle to the knee and hip joints reducing long distance running economy (Sanno et al., 2018).

Foot pronation and joint impact forces have been proposed as predictors of RRI (Nigg, 2011; Brund et al., 2017). Gait retraining programs (Bowser et al., 2018) and real time feedback studies (Yong et al., 2018) evaluated potential factors contributing to impact RRI, such as peak tibial shock (peak vertical acceleration), and average and peak loading rates. Conflicting opinions concerning foot pronation as a risk factor has reported for neutral shoes (Nielsen et al., 2014), and standard versus motion control shoes (Malisoux et al., 2016). The contradicting results may be explained in part by different runners’ experience, running footwear preferences, and different study designs. Bertelsen et al. (2017) proposed a framework to analyze the etiology of RRI, whereby cumulative load exceeding a maximum load capacity would trigger injury. Studies have revealed alterations in gait symmetry, joint stability and power contribution in competitive long distance runners (Hanley and Tucker, 2018; Sanno et al., 2018; Hoenig et al., 2019). The literature presents multiple factors contributing to RRI in competitive athletes, however, few studies consider the effects on recreational runners, who are the majority of the running population (Knechtle et al., 2018; Vitti et al., 2019). Foot pronation has been reported as a predictor of altered joint kinetics and running related injuries (Nigg, 2011; Brund et al., 2017), however, a quantitative measure between the clinical FPI (a score that measures pronation) and joint kinetics has not been presented to date.

Thus, the aim of this study was to investigate the changes of foot posture, joint kinematics, joint moments and joint contact forces in the lower extremity following a 5 k treadmill run in recreational runners. We present the FPI and its relation to lower limb kinetics pre and post-5 k running. It is hypothesized that (1) joint kinematics, joint moments and joint contact forces in the lower extremity will change post-5 k running, and (2) the FPI will quantify changes in joint kinetics following mid distance running.

## Materials and Methods

### Participants

Twenty recreational male heel strike runners (25.8 ± 1.6 years, 67.8 ± 5.3 kg, 1.73 ± 0.05 m) participated in this study, consistent with previous running studies (Hanley and Tucker, 2018; Sanno et al., 2018; Hoenig et al., 2019). The inclusion criteria was participants would have over ground or treadmill running history with an average distance of 30 km per week and preference using typical running shoes. Participants were free from lower extremity disorders and injuries. Foot deformities, such as hallux valgus, over pronation or supination, pes planus, and pes cavus, were excluded during recruitment. Written consent was obtained prior to the test. Ethics was approved from the Human Ethics Committee at Ningbo University (RAGH20161208).

### Experimental Protocol

Baseline data (pre 5 k run) were collected with the participant standing barefoot (static) followed by running barefoot on the over ground runway at their self-selected speed. This included a static foot posture assessment, static marker positions, dynamic marker trajectories, ground reaction force and surface electromyography (EMG). The assessment of foot posture was performed following the established FPI (Redmond et al., 2006), including six observations from the (1) talar palpation, (2) malleoli, (3) inversion/eversion of calcaneus in the rearfoot, (4) talonavicular joint, (5) medial longitudinal arch, and (6) forefoot abduction/adduction to define foot postures in multiple planes and anatomical segments. An eight-camera motion capture system (Vicon Metrics Ltd., Oxford, United Kingdom) was used to track the marker trajectories at 200 Hz, and an in-ground force plate (AMTI, Watertown, MA, United States) was utilized to record the ground reaction force at 1000 Hz. The force plate was located in the middle of an over ground runway. A 37-marker set was used for all participants during the test, which has been validated in previous studies (Hamner and Delp, 2013; Rajagopal et al., 2016). Surface electromyography (EMG) signals were recorded via a EMG system (Delsys, Boston, MA, United States) for muscle activities, including rectus femoris (RF), vastus lateralis (VL), vastus medialis (VM), biceps femoris (BF), semitendinosus (ST), tibialis anterior (TA), medial gastrocnemius (MG), and lateral gastrocnemius (LG).

After warm-up and lab familiarization, the FPI was evaluated and recorded as scores (from -2 to 2 per item). The total score would be classed as high supination (-12), supination (-5), neutral (0), pronation (5), and high pronation (12) while static barefoot standing with shoulders’ width apart (Redmond et al., 2006). Data of marker trajectories and ground reaction force from two static and five running trials were collected of the right foot striking the force plate. After the baseline test, participants ran 5 k on the treadmill at their self-selected speed (which were recorded in the range of 10–12 km/h) using participants’ own typical running shoes. This was not chosen to elicit fatigue but elicit submaximal effort (Hanley and Tucker, 2018). The post-5 k test started within 5 min of finishing the treadmill run, following the same protocols as the baseline test (with participants barefoot).

### Musculoskeletal Model

An updated version of the original OpenSim musculoskeletal model (Delp et al., 2007), which included the patella (DeMers et al., 2014), was used for this study. This model included the torso and lower extremity, which had six degrees of freedom at the pelvis, a ball-and-socket joint with three degrees of freedom at the hip, pin joints at the ankle, subtalar and metatarsophalangeal joints. A non-frictional patella articulated with the femur and prescribed by the knee angle was also added to direct the quadriceps force, wrapping around the patella and attaching to the tibial tuberosity (DeMers et al., 2014). The default model included a hinge joint for flexion-extension of the knee, and was extended to include abduction-adduction motion based on a previous study (Meireles et al., 2017).

Data processing was performed in OpenSim v3.3 as per the established workflow (Delp et al., 2007). Marker trajectories and ground reaction forces were low pass filtered at 6 Hz with a zero-phase fourth order Butterworth filter. The model was firstly scaled to each participant’s anthropometric measures collected from static marker positions and body mass. Muscle insertion points and moment arms were scaled to match each participants’ segment lengths (DeMers et al., 2014). The “*Inverse kinematics*” (IK) algorithm minimized errors between virtual markers in the model and experimental marker trajectories to compute joint angles, and “*Inverse Dynamics*” (ID) was performed to compute joints moment (Delp et al., 2007).

Muscle forces were previously reported as the main factors affecting joint contact forces (DeMers et al., 2014; Lerner et al., 2015; Lerner and Browning, 2016). The “*Static Optimization*” (SO) with weighted factors was employed to compute muscle activation and forces, which improves the accuracy of joint contact force prediction (DeMers et al., 2014; Lerner and Browning, 2016). Following previously established protocols to reduce prediction errors (Lerner et al., 2015; Lerner and Browning, 2016), the weighting factors for muscles were set at 1.5 for the gastrocnemius, 2 for the hamstrings and 1 for other muscles in this study. The contact forces to the hip, knee and ankle joints in the anterior/posterior (x), superior/inferior (y), and medial/lateral (z) directions were computed using “*Joint Reaction*” (JR) analysis for the femur, tibia and talus, respectively.

### Model Validation

Muscle electromyography (EMG) signals were used to validate model-simulated muscle activations (Supplementary Material 1), which included the rectus femoris (RF), vastus lateralis (VL), vastus medialis (VM), biceps femoris (BF), semitendinosus (ST), tibialis anterior (TA), medial gastrocnemius (MG), and lateral gastrocnemius (LG). Joint kinematics, joint kinetics, and joint contact force were compared with previous literature.

### Data and Statistical Analysis

A simulation of stance phase from right heel strike to toe off was analyzed in this study. Variables included FPI scores, joint angles, joint moments and joint contact force in the anterior/posterior (**ant-post**) (x), superior/inferior (**sup-inf**) (y), and medial/lateral (**med-lat**) (z) directions during pre-5 k and post-5 k tests. For the time sequential kinematics, kinetics and contact force data, raw data from five trials of each participant were interpolated to 50 in data length to represent stance, and averaged for each participant for statistics. The joint moments (flexion/extension, adduction/abduction, and internal/external rotation moments of the hip, flexion/extension and adduction/abduction moments of the knee, dorsi/plantar flexion moment of the ankle, inversion/eversion moment of subtalar) and contact forces were normalized to body mass (Nm/kg) for moments and body weight (xBW) for contact forces, respectively. Peak values of joint moments and joint contact forces were selected for statistics. Previously published studies concerning knee **sup-inf** contact force showed similar patterns with vertical ground reaction force (Steele et al., 2012; Gerus et al., 2013; Knarr and Higginson, 2015), thus this study calculated the vertical instantaneous loading rate (VILR) (unit: xBW/%stance) of **sup-inf** knee contact force using an established protocol (Ueda et al., 2016), to provide extra loading information to the knee joint. Stance was divided into three sub-phases as per previous studies (Novacheck and Tom, 1998; Dugan and Bhat, 2005), including initial contact (0∼50%), mid stance (∼50%∼), and push off (50∼100%).

Data normality was checked prior to statistical analysis. A paired sample *t*-test was performed to analyze the difference in FPI scores, running speed, contact times, peak joint moments and joint contact forces. Due to the one-dimensional (1D) time-varying characteristics of joint kinematics, joint moments and joint contact force (Pataky, 2010; Pataky et al., 2015), the open source Statistical Parametric Mapping 1D package (SPM1D), which relies on Random Vector Field theory to account for data variability, was utilized for the statistical analysis. All statistical analyses were performed in MATLAB R2018a (The MathWorks, MA, United States), with significance level set at *p* < 0.05.

## Results

### Foot Posture and Gait Parameter Changes

The FPI scores measured pre-5 k and post-5 k running showed significant increase toward pronation. The pre and post-5 k running speeds measured during the gait test were found to be ∼3.1 m/s on average. Participants were instructed to run 5 k at their self-selected speed, and actual speeds were recorded in the range of 10–12 km/h (2.8–3.3 m/s), with completion time between 25.3 and 29.7 min. A statistically significant increase of running speed was observed post-5 k running but stance times remained unchanged (Table 1).

**Table 1:** FPI scores, speed, and contact time (Mean ± SD [95% Confidence Interval]).

Variables	Pre-5 k [95% CI]	Post-5 k [95% CI]	*p*-value
FPI scores	1.7 ± 1.84 [0.84, 2.56]	7.3 ± 1.87 [6.43, 8.17]	<0.001
Speed (m/s)	3.068 ± 0.128 [3.0, 3.13]	3.137 ± 0.152 [3.07, 3.21]	0.007
Contact time (s)	0.253 ± 0.023 [0.242, 0.263]	0.249 ± 0.027 [0.236, 0.262]	0.230


### Hip Joint

**FIGURE 1:**
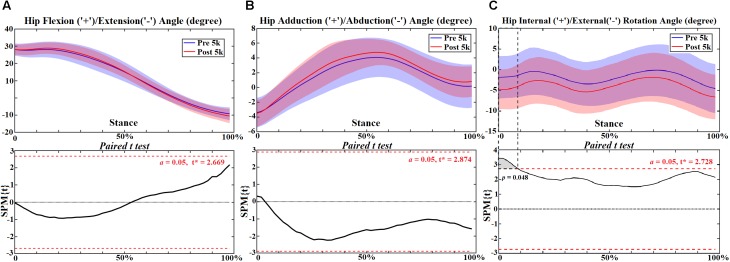
The hip joint angles **(A–C)** during stance with statistics (spm{t}) from spm1d (“+” and “-” represent directions).

**FIGURE 2:**
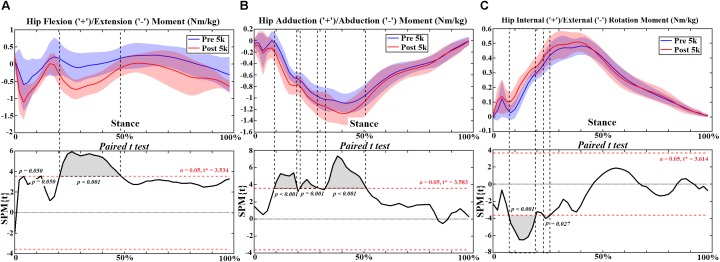
The hip moments **(A–C)** during stance with statistics (spm{t}) from spm1d (“+” and “-” represent directions).

**FIGURE 3:**
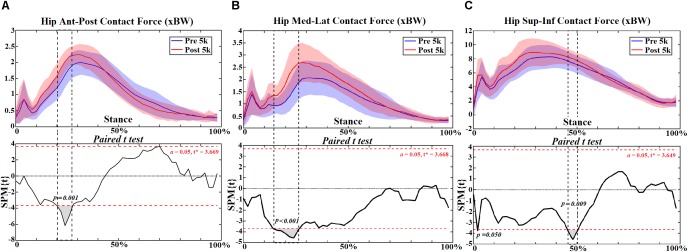
The hip contact forces **(A–C)** during stance with statistics (spm{t}) from spm1d (“+” and “-” represent directions).

At the hip joint during post-5 k running, external rotation angle increased at 0–10% (*p* = 0.048) (Figure 1) and rotation moment increased at 10%–20% (*p* < 0.001) and 26%–28% (*p* = 0.027) (Figure 2C). Increased extension moment was observed across stance at 6% (*p* = 0.050), 14% (*p* = 0.050) and 24%–50% (*p* < 0.001) (Figure 2A). Abduction moment increased at 12%–20% (*p* < 0.001), 24%–30% (*p* = 0.001), and 36%–52% (*p* < 0.001), respectively (Figure 2B). The contact force increased in the ant-post-direction at 22–28% (*p* = 0.001) (Figure 3A), in the med-lat direction at 16–28% (*p* < 0.001) (Figure 3B), and in the sup-inf direction at 48–52% (*p* = 0.009) (Figure 3C). Peak hip moments and contact force are presented (Table 2), with increased peak hip extension moment (*p* = 0.024) and abduction moment (*p* < 0.001), and peak hip contact force in the ant-post (*p* = 0.001), med-lat (*p* < 0.001), and sup-inf (*p* = 0.002) directions during post-5 k running.

**Table 2:** The peak hip moments and joint contact forces in the ant-post, med-lat, and sup-inf directions during stance (Mean ± SD [95% Confidence Interval]).

Variables	Pre-5 k [95% CI]	Post-5 k [95% CI]	*p*-value
Ext moment (Nm/kg)	1.13 ± 0.39 [0.95, 1.31]	1.35 ± 0.44 [1.15, 1.56]	0.024
Abd moment (Nm/kg)	1.14 ± 0.17 [1.06, 1.22]	1.3 ± 0.21 [1.20, 1.40]	<0.001
Rot moment (Nm/kg)	0.51 ± 0.06 [0.48, 0.54]	0.52 ± 0.07 [0.50, 0.56]	0.087
Ant-post contact force (xBW)	2.10 ± 0.39 [1.91, 2.28]	2.36 ± 0.3 [2.21, 2.50]	0.001
Med-lat contact force (xBW)	2.4 ± 0.72 [2.06, 2.74]	3.0 ± 0.81 [2.62, 3.38]	<0.001
Sup-inf contact force (xBW)	8.76 ± 1.61 [8.0, 9.5]	9.71 ± 1.65 [8.9, 10.48]	0.002


### Knee Joint

**FIGURE 4:**
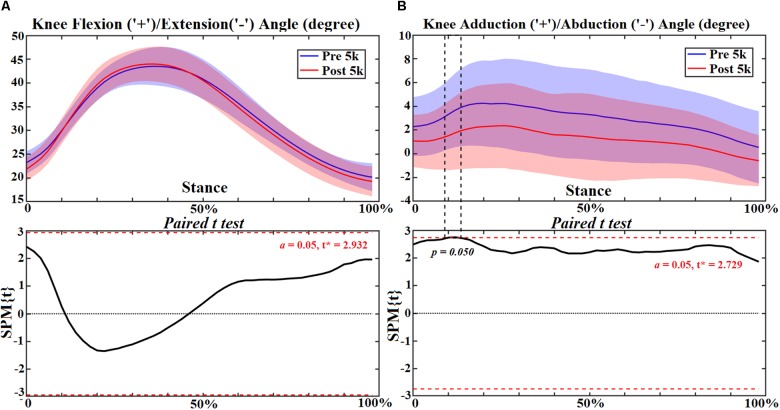
The knee joint angles **(A,B)** during stance with statistics (spm{t}) from spm1d (“+” and “-” represent directions).

**FIGURE 5:**
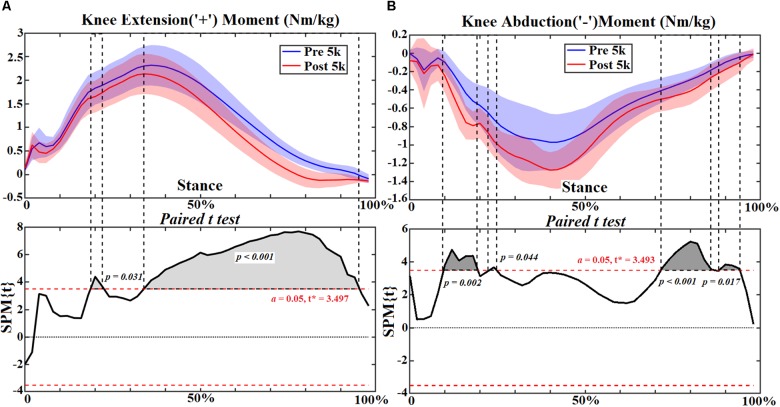
The knee joint moments **(A,B)** during stance with statistics (spm{t}) from spm1d (“+” and “-” represent directions).

**FIGURE 6:**
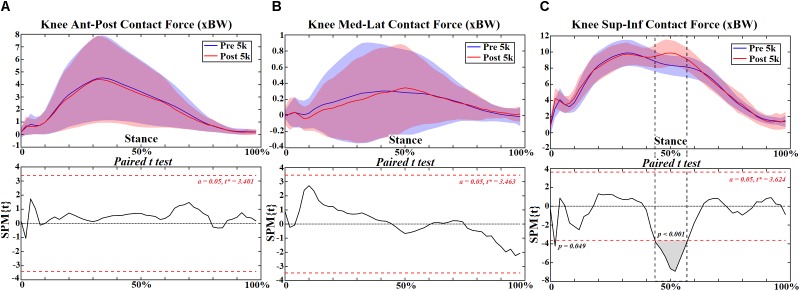
The knee joint contact forces **(A–C)** during stance with statistics (spm{t}) from spm1d (“+” and “-” represent directions).

At the knee joint, flexion angle showed no change (Figure 4A) but adduction reduced at 12–14% (*p* = 0.050) of stance (Figure 4B). However, reduced extension moment was observed at 22–24% (*p* = 0.031) and 36–96% (*p* < 0.001) (Figure 5A). Increased knee abduction moment was observed at 12–20% (*p* = 0.002) and 26% (*p* = 0.044) during initial contact, and at 74–88% (*p* < 0.001) and 92–96% (*p* = 0.017) during push off, respectively (Figure 5B). The knee contact force increased during mid stance (46–58%, *p* < 0.001) in the sup-inf direction (Figure 6C) but no significance in other directions (Figure 6A,B). Table 3 presents the peak knee joint extension (*p* = 0.001) and abduction (*p* = 0.002) moments, and the VILR (*p* < 0.001) and peak values of sup-inf (*p* = 0.005) knee contact force.

**Table 3:** The peak knee moments and joint contact forces in the ant-post, med-lat, and sup-inf directions (Mean ± SD [95% Confidence Interval]).

Variables	Pre-5 k [95% CI]	Post-5 k [95% CI]	*p*-value
Ext moment (Nm/kg)	2.33 ± 0.44 [2.12, 2.53]	2.15 ± 0.44 [1.94, 2.35]	0.001
Abd moment (Nm/kg)	0.99 ± 0.31 [0.85, 1.14]	1.11 ± 0.28 [0.97, 1.23]	0.002
VILR (BW/Stance%)	100.1 ± 33.04 [84.65, 115.58]	131.73 ± 28.83 [118.24, 145.22]	<0.001
Ant-post contact force (xBW)	4.95 ± 3.0 [3.55, 6.35]	4.74 ± 3.3 [3.19, 6.28]	0.46
Med-lat contact force (xBW)	0.63 ± 0.34 [0.47, 0.80]	0.58 ± 0.4 [0.39, 0.77]	0.52
Sup-inf contact force (xBW)	10.12 ± 1.58 [9.38, 10.86]	10.88 ± 1.49 [10.18, 11.58]	0.005


**FIGURE 7:**
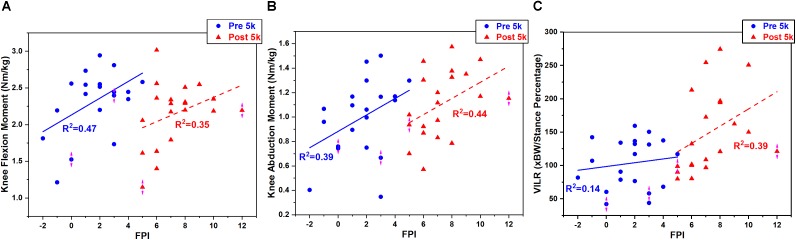
The correlation of peak knee joint loadings (**A,** flexion moment; **B,** abduction moment; **C,** vertical loading rate) with FPI.

Correlation between FPI scores pre-5 k and post-5 k with peak knee flexion moment, peak knee abduction moment and VILR are presented in Figure 7. There was a moderate correlation between FPI and peak knee flexion moment (0.35–0.47), during pre- and post-5 k treadmill running (Figure 7A). The correlation between FPI and peak knee abduction moment was also moderate (0.39–0.44), during pre and post-5 k (Figure 7B). Interestingly, the correlation between FPI and VILR was only moderate post-5 k (0.39) (Figure 7C).

### Ankle Joint

**FIGURE 8:**
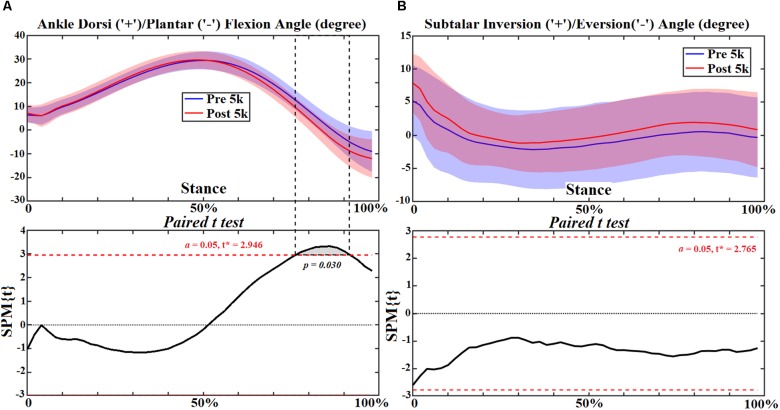
The ankle and subtalar joint angles **(A,B)** during stance with statistics (spm{t}) from spm1d (“+” and “-” represent directions).

**FIGURE 9:**
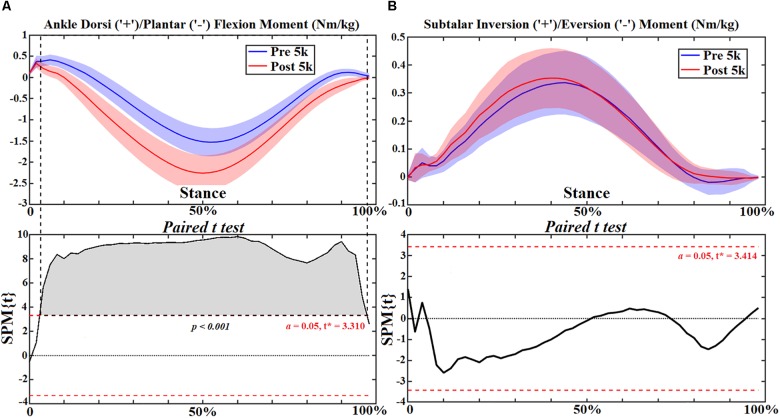
The ankle and subtalar joint moments **(A,B)** during stance with statistics (spm{t}) from spm1d (“+” and “-represent directions).

**FIGURE 10:**
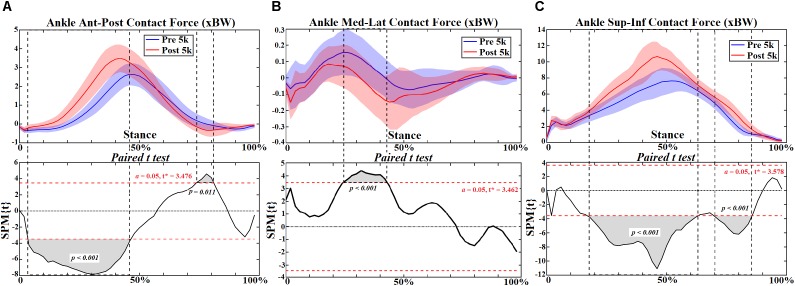
The ankle joint contact forces **(A–C)** during stance with statistics (spm{t}) from spm1d (“+” and “-” represent directions).

**FIGURE 11:**
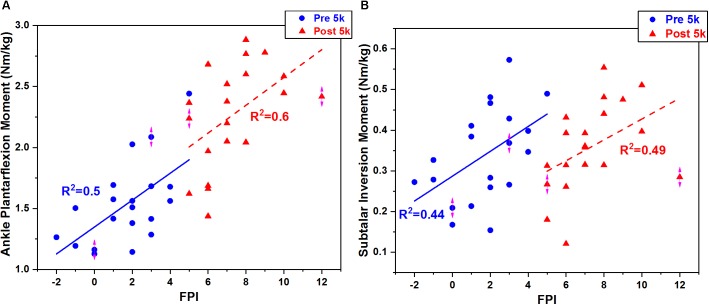
The correlation of peak ankle **(A)** and subtalar **(B)** moments with FPI.

At the ankle joint increased plantarflexion was observed during push off at 80–92% (*p* = 0.030) (Figure 8A), and the plantarflexion moment increased at 6–98% (*p* < 0.001) during stance (Figure 9A). However, the subtalar joint eversion angle (Figure 8B) and subtalar moment (Figure 9B) showed no change. The ankle contact force in the ant-post direction increased at 6%-48% (*p* < 0.001) but decreased at 76–82% (*p* = 0.011) (Figure 10A). The med-lat ankle contact force decreased at 28–44% (*p* < 0.001) (Figure 10B). The sup-inf ankle contact force increased at 20–64% (*p* < 0.001) and 72–86% (*p* < 0.001) (Figure 10C), respectively. Table 4 presents the peak ankle plantarflexion moment (*p* < 0.001), ankle contact force in the ant-post (*p* < 0.001) and sup-inf (*p* < 0.001) directions. The correlations between FPI and peak ankle moment (0.5–0.6) and subtalar moment (0.44–0.49) were moderate in both cases (Figure 11A,B).

**Table 4:** The peak ankle and subtalar moments and ankle joint contact forces in the ant-post, med-lat, and sup-inf directions (Mean ± SD [95% Confidence Interval]).

Variables	Pre-5 k [95% CI]	Post-5 k [95% CI]	*p*-value
Plantarflexion moment (Nm/kg)	1.54 ± 0.34 [1.38, 1.39]	2.26 ± 0.43 [2.05, 2.47]	<0.001
Inversion moment (Nm/kg)	0.34 ± 0.12 [0.29, 0.39]	0.36 ± 0.11 [0.31, 0.41]	0.350
Ant-post contact force (xBW)	2.77 ± 0.62 [2.48, 3.06]	3.71 ± 0.66 [3.41, 4.02]	<0.001
Med-lat contact force (xBW)	0.25 ± 0.11 [0.20, 0.30]	0.27 ± 0.12 [0.22, 0.33]	0.410
Sup-inf contact force (xBW)	8.09 ± 1.55 [7.36, 8.82]	11.24 ± 1.76 [10.4, 12.06]	<0.001


## Discussion

The findings in this study suggest that joint moments and joint contact forces in the lower extremity are altered with increased foot pronation following 5 k running. Specifically, hip joint moments and hip contact force increased during stance. Knee joint extension moment decreased but abduction moment increased, and sup-inf contact force increased during mid stance. Ankle plantarflexion moment increased throughout stance, and ankle contact force increased in the ant-post and sup-inf directions but decreased in the med-lat direction. The FPI was found to correlate moderately with knee and ankle moments pre- and post-5 km running.

The human foot attenuates shock at the arch during weight bearing in stance. Due to repetitive loading from prolonged running activities, reduced arch height and pronated foot posture are reported in long distance runners (Fukano et al., 2018; Mei et al., 2018), which is consistent with the increased foot pronation assessed using the FPI in this study. Foot pronation may be associated with several RRI, which remain a conflicting issue in the biomechanics community. High arch runners present with higher incidence of ankle injuries, in contrast low arch runners exhibit more knee injuries (Williams et al., 2001), specifically the medial tibia stress syndrome among lower arch and pronated foot runners (Bennett et al., 2001). Greater knee abduction moment has been reported during walking and running in athletes with a low foot arch (Powell et al., 2016). This is consistent with the current study that showed a moderate correlation between FPI (pronated with low arch) and peak abduction moment. It should be acknowledged that participants in this study wore their preferred shoe design and this was not controlled for. Shoe design has been shown to influence pronation including motion control shoes (Malisoux et al., 2016), maximal, neutral, and minimal shoes (Mei et al., 2014; Pollard et al., 2018; Xiang et al., 2018). Footwear design or wearing no shoes at all may influence the motor control system during running (Feng and Song, 2017; Santuz et al., 2017).

Stance contact time after 5 k running was consistent with a recent study of intersegmental work contribution during a prolonged run (Sanno et al., 2018). However, the average speed of runners in this study was ∼3.1 m/s, which was slower than the study of exhaustive maximal 10 k treadmill running (Hanley and Tucker, 2018) reported as ∼4.7 m/s. This is likely due to runners in that study being competitive compared to the recreational class of the runners in the present study. Comparison with other recreational running studies revealed speeds of 3.3–3.4 m/s (Hoenig et al., 2019) and 3.2 m/s (Chan-Roper et al., 2012), which was consistent with our findings.

Sagittal and coronal hip kinematics remained unchanged post-5 k running in this study. This was consistent with a 10 k treadmill study of recreational runners at the same 5 k mark (Sanno et al., 2018). In overuse injuries in recreational runners it has been reported that hip flexor, abductor and external rotator muscle strength is reduced (Niemuth et al., 2005; Luedke et al., 2015; Kollock et al., 2016). The reduced muscles lead to an imbalance of the hip joint moments and the net result is increased extension, abduction and internal rotation moments. This is consistent with the current study where we found increased extension moment, abduction moment and internal rotation moment during the initial contact of stance.

The sup-inf hip contact force from this study was 8.8–9.7 BW at 3.1 m/s, which was consistent with a previous running study that reported hip contact forces of 9.47 BW when running at 3.05 m/s (Giarmatzis et al., 2015). It should be noted that the hip contact force in the current study further highlighted that sup-inf contact force increased during mid stance, whereas the med-lat and ant-post contact forces only increased during initial contact. Further, the pattern of sup-inf knee contact force was similar to the vertical ground reaction force, which is consistent with previous studies (Steele et al., 2012; Gerus et al., 2013; Knarr and Higginson, 2015).

Knee flexion and adduction kinematics and joint moments were consistent in profile and magnitude range with previous running studies (Hamner et al., 2010; Bonacci et al., 2013; Hamner and Delp, 2013). Simulated knee crossing muscle activation patterns (vastus lateralis, rectus femoris and vastus medialis) were in good temporal agreement with EMG signals recoded in our study (see Supplementary Material). Significantly decreased knee extension moment was observed from mid stance to push off during post-5 k running, which may be partly explained by the weak extensor muscles reported for recreational runners (Kollock et al., 2016).

The FPI was found to partly explain the knee flexion and knee abduction moments both pre and post-5 k running. Specifically, as the foot pronates knee abduction increases. This is interesting since increased knee abduction (or reduced knee adduction) has been associated with reduced medial knee loading in people who walk with increased foot pronation (Levinger et al., 2013). However, in contrast increased pronation has also been reported to be associated with medial loading and tibia stress (Barnes et al., 2008; Levinger et al., 2010) and everted foot kinematics during locomotion (Levinger et al., 2012). This suggests that foot pronation plays a role in medial knee joint loading and should not be too over pronated or supinated.

Ankle joint kinematics at heel strike and toe off during pre- 5 k and post-5 k were consistent with recent studies (Reenalda et al., 2016; Sanno et al., 2018) showing similar profiles and range of motion. The subtalar joint angle and moment patterns were unchanged post-5 k running, however, the single calcaneus marker used in this study may not be suited for dynamic subtalar joint motions in the frontal plane and should be considered as a limitation (Wang and Gutierrez-Farewik, 2011; Fischer et al., 2017). Our study showed increased plantarflexion during push off and plantarflexion joint moment throughout stance post-5 k running. One item exhibited from the FPI in this study was increased calcaneus eversion at the subtalar joint post-5 k running. This is consistent with a study that reported subtalar over eversion was found to enlarge the plantar flexors and tibialis anterior muscles (Wang and Gutierrez-Farewik, 2011). Further, increased plantar flexor muscles and tibialis anterior (dorsiflexor) may contribute to increased ankle contact forces. This is consistent with the increased ankle contact force observed in this study.

## Conclusion

This study presents an investigation of the changes in foot posture, joint kinematics, joint moments and joint contact forces in the lower extremity following a 5 k treadmill run in 20 participants. A relationship between knee and ankle joint loading and FPI was developed. It was found that hip joint moments and contact forces increased during initial foot contact following 5 k running. Knee abduction moment and superior-inferior knee contact force increased, whereas the knee extension moment decreased. Ankle plantarflexion moment and ankle contact forces increased during stance. A useful finding was that the FPI was moderately correlated with peak knee and ankle moments. The FPI showed that recreational male runners presented increased static foot pronation after 5 k treadmill running. These findings suggest that following mid distance running change in foot pronation may be an early indicator of increased lower limb joint loading. Furthermore, the FPI may be used to quantify the changes in knee and ankle joint moments. Specifically, increase in FPI leads to an increase in knee flexion moment, knee abduction moment, ankle plantarflexion moment and subtalar inversion moment.

## Data Availability

The raw data supporting the conclusions of this manuscript will be made available by the authors, without undue reservation, to any qualified researcher.

## Ethics Statement

This study was approved by the Ethical Committee in the Research Academy of Grand Health, Ningbo University (RAGH20161208).

## Author Contributions

QM, YG, and JF conceived and designed this study. QM and LX conducted the test, collected, and analyzed the data. QM, YG, JB, and JF prepared the manuscript. QM, YG, LX, JB, and JF commented, revised the manuscript, and all approved for the submission.

## Conflict of Interest Statement

The authors declare that the research was conducted in the absence of any commercial or financial relationships that could be construed as a potential conflict of interest.
