# Supplementary material for: Foot Pronation Contributes to Altered Lower Extremity Loading After Long Distance Running
Source: Front Physiol. 2019 May 22;10:573. doi: 10.3389/fphys.2019.00573 (PMC6540596; doi:10.3389/fphys.2019.00573)
Supplement: Supplementary file 1 [file Data_Sheet_1.PDF]

Supplementary Material 1 Model validation

The model validation was performed via comparing the surface EMG activity with model simulated muscle activation. Surface EMG measurement was conducted using wireless 16-channel Delsys system (Delsys, Boston, Massachusetts, US) at 1000Hz. Electrodes were placed on the rectus femoris (RF), vastus lateralis (VL), vastus medialis (VM), biceps femoris (BF), semitendinosus (ST), tibialis anterior (TA), medial gastrocnemius (MG) and lateral gastrocnemius (LG).

Apart from measured EMG activity pre 5k and post 5k run, the EMG of above muscles was recorded while participants performing the maximum voluntary contraction (MVC). The root mean square (RMS) calculation algorithm (**Equation 1**) was used for MVC and running trials in the Delsys EMGworks Analysis software, in which S represents window length (points) and f(s) represents data in the window.

$$\text{RMS} = \left(\frac{1}{S}\sum_{1}^{S} f^{2}(s)\right)^{\frac{1}{2}} \qquad (\mathbf{1})$$

Model simulated activation from OpenSim were reported on a scale from zero to one, with zero representing non-activation and one representing maximum activation. The EMG activities of pre 5k and post 5k running stance trials were normalized to a zero-one scale using the following **equation** (**2**),

$$\text{EMG activity} = \frac{trial\ RMS\ amplitude\ value}{MVC\ RMS\ amplitude\ value} \qquad (\mathbf{2})$$

See below figures for the validation of EMG and simulated activation. These results have been compared with findings of Hamner et al (2010), Hamner et al (2013) and Rajagopal et al (2016).

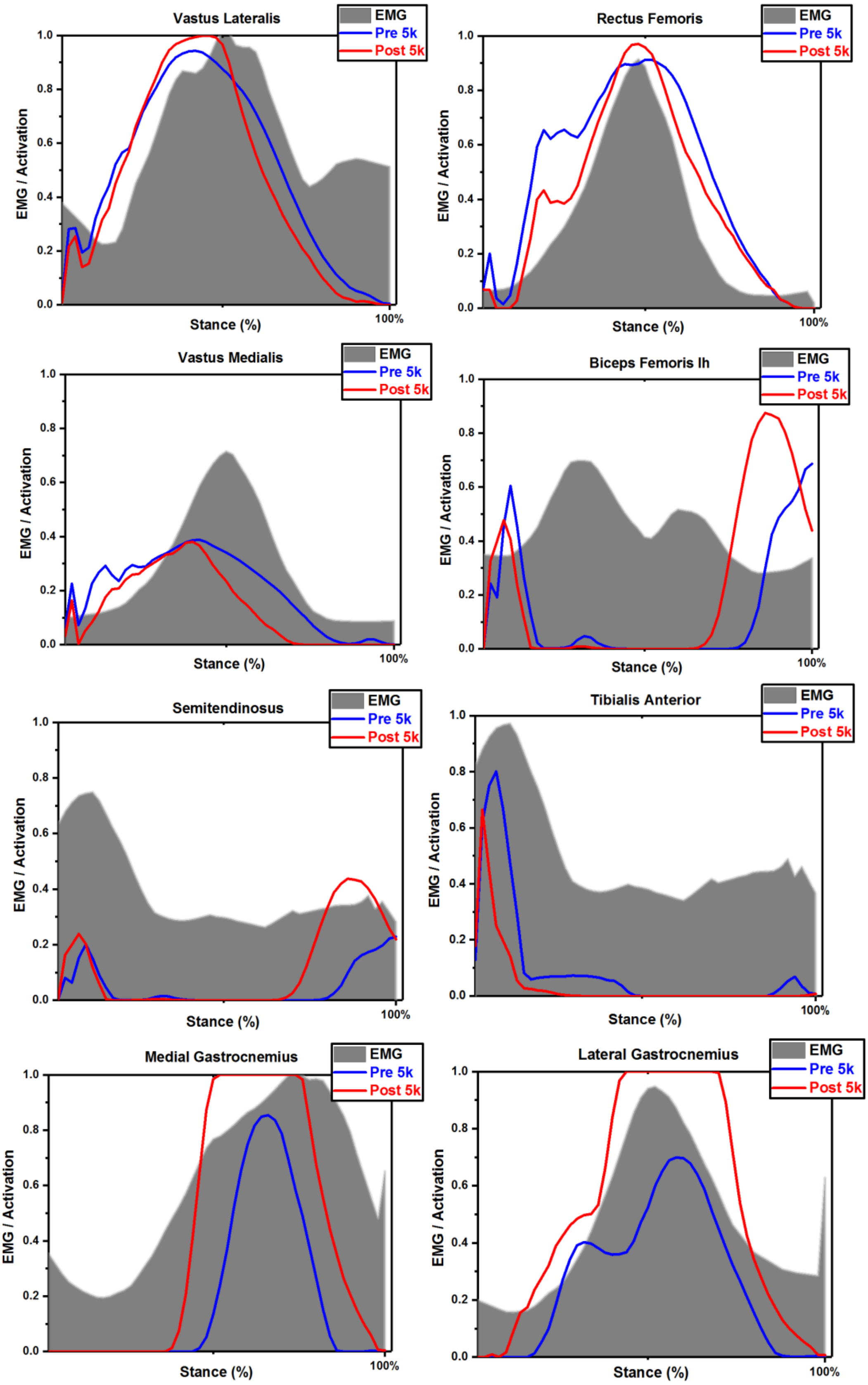

Vastus Lateralis
EMG
Pre 5k
Post 5k
EMG / Activation
1.0
0.8
0.6
0.4
0.2
0.0
Stance (%)
100%
Rectus Femoris
EMG
Pre 5k
Post 5k
EMG / Activation
1.0
0.8
0.6
0.4
0.2
0.0
Stance (%)
100%
Vastus Medialis
EMG
Pre 5k
Post 5k
EMG / Activation
1.0
0.8
0.6
0.4
0.2
0.0
Stance (%)
100%
Biceps Femoris lh
EMG
Pre 5k
Post 5k
EMG / Activation
1.0
0.8
0.6
0.4
0.2
0.0
Stance (%)
100%
Semitendinosus
EMG
Pre 5k
Post 5k
EMG / Activation
1.0
0.8
0.6
0.4
0.2
0.0
Stance (%)
100%
Tibialis Anterior
EMG
Pre 5k
Post 5k
EMG / Activation
1.0
0.8
0.6
0.4
0.2
0.0
Stance (%)
100%
Medial Gastrocnemius
EMG
Pre 5k
Post 5k
EMG / Activation
1.0
0.8
0.6
0.4
0.2
0.0
Stance (%)
100%
Lateral Gastrocnemius
EMG
Pre 5k
Post 5k
EMG / Activation
1.0
0.8
0.6
0.4
0.2
0.0
Stance (%)
100%

Reference

Hamner, S.R., Seth, A., Delp, S.L., 2010. Muscle contributions to propulsion and support during running. J. Biomech. 43, 2709–2716. https://doi.org/10.1016/j.jbiomech.2010.06.025

Hamner, S.R., Delp, S.L., 2013. Muscle contributions to fore-aft and vertical body mass center accelerations over a range of running speeds. J. Biomech. 46, 780–787. https://doi.org/10.1016/j.jbiomech.2012.11.024

Rajagopal A, Dembia CL, DeMers MS, et al. 2016. Full-Body Musculoskeletal Model for Muscle-Driven Simulation of Human Gait. IEEE Trans. Biomed. Eng. 63, 2068–2079. doi:10.1109/TBME.2016.2586891
